# 3D Reconstruction of Lipid Droplets in the Seed of *Brassica napus*

**DOI:** 10.1038/s41598-018-24812-2

**Published:** 2018-04-26

**Authors:** Yongtai Yin, Liangxing Guo, Kang Chen, Zhenyi Guo, Hongbo Chao, Baoshan Wang, Maoteng Li

**Affiliations:** 1 Department of Biotechnology, College of Life Science and Technology, Huazhong University of Science and Technology, Wuhan, 430074 China; 2 College of Life Science, Shandong Normal University, Jinan, 250000 China; 3 Hubei Key Laboratory of Economic Forest Germplasm Improvement and Resource Comprehensive Utilization, Hubei Collaborative Innovation Center for the Characteristic Resources Exploitation of Dabie Mountains, Huanggang Normal University, Huanggang, 438000 China

## Abstract

Rapeseed is one of the most important and widely cultured oilseed crops for food and nonfood purposes worldwide. Neutral lipids are stored in lipid droplets (LDs) as fuel for germination and subsequent seedling growth. Most of the LD detection in seeds was still in 2D levels, and some of the details might have been lost in previous studies. In the present work, the configuration of LDs in seeds was obtained by confocal imaging combined with 3D reconstruction technology in *Brassica napus*. The size and shape of LDs, LD numbers, cell interval spaces and cell size were observed and compared at 3D levels in the seeds of different materials with high and low oil content. It was also revealed that different cells located in the same tissue exhibited various oil contents according to the construction at the 3D level, which was not previously reported in *B. napus*. The present work provides a new way to understand the differential in cell populations and enhance the seed oil content at the single cell level within seeds.

## Introduction

Internal structural visualization and quantification of plant tissue are essential to fully understand the structure-function relationship, dynamic processes and evolution in plants^1–3^. The internal structure of plant tissues can be visualized by microscopy technology at the two-dimensional (2D) level by histologic section^4–8^. However, the histological sectioning of plant tissue is laborious, and some tiny details in the slicing process are frequently lost^9,10^. With the availability of new imaging techniques, there are exciting new possibilities to visualize tiny structures at the three-dimensional (3D) level with some interesting new approaches^9,11–13^.

3D image analysis and modeling in plants is in its infancy and holds great promise to uncover further mechanistic principles underlying plant growth and development^14^. 3D reconstruction technology has been successfully applied to observe brain structures in mammals^11,15–17^. Scientists acquired micrometer-scale tomography of a centimeter-sized whole mouse brain and a 3D structural data set of a stained whole mouse brain by micro-optical sectioning tomography system^12^. In recent years, 3D imaging has become an increasingly popular and promising approach to investigate plant growth and development^18–22^. Many technologies have been applied in 3D reconstruction, such as X-ray-based computed tomography (CT)^1^, nuclear magnetic resonance (NMR)^23–25^, and micro-optical sectioning tomography (MOST)^12^. Confocal laser scanning microscopy (CLSM) has been effectively used to visualize cellular structures and to produce 3D images of larger anatomical structures of plant tissues^10^. With the rapid advance of 3D reconstruction technology, large sets of imaging data processing software such as AMIRA, 3DCellAtlas and MorphoGraphX allow the simultaneous integration of regulatory components within these individual cells in *Arabidopsis*^2^.

As one of the most important and widely cultured oilseed crops, studies on lipid distribution and arrangement in rapeseed could help to understand the biological mechanism of lipid accumulation and would benefit breeding to increase the oil content. Seed was considered the major organ to store abundant levels of oil in lipid droplets (LDs)^26^. LDs are lipid storage organelles with a core of neutral lipids covered by a phospholipid monolayer and oleosins in most eukaryotes organisms^27^. LDs were of interest to scientists because of their relationship with levels of oil content^28–30^. Additionally, the structural organization of seed LDs could affect the oil extractability of rapeseed^31^. In previous studies, LD size in seeds was usually observed at the 2D level^32–34^. The application of 3D image reconstruction technology in plants has practical significance. Recently, the LD fusion process was successfully observed by 3D reconstruction technology^35^. The neutral lipid shape and their distribution at the 3D level have been previously reported.

In this study, the 3D structure of LDs and the cell membrane was constructed to analyze the shapes and configurations of lipids more accurately in seeds of *B. napus*, which will provide a new way to understand the lipid distribution characteristics inside seeds.

## Results

### CLSM imaging and three-dimensional reconstruction of lipid and cell shape

**Figure 1:**
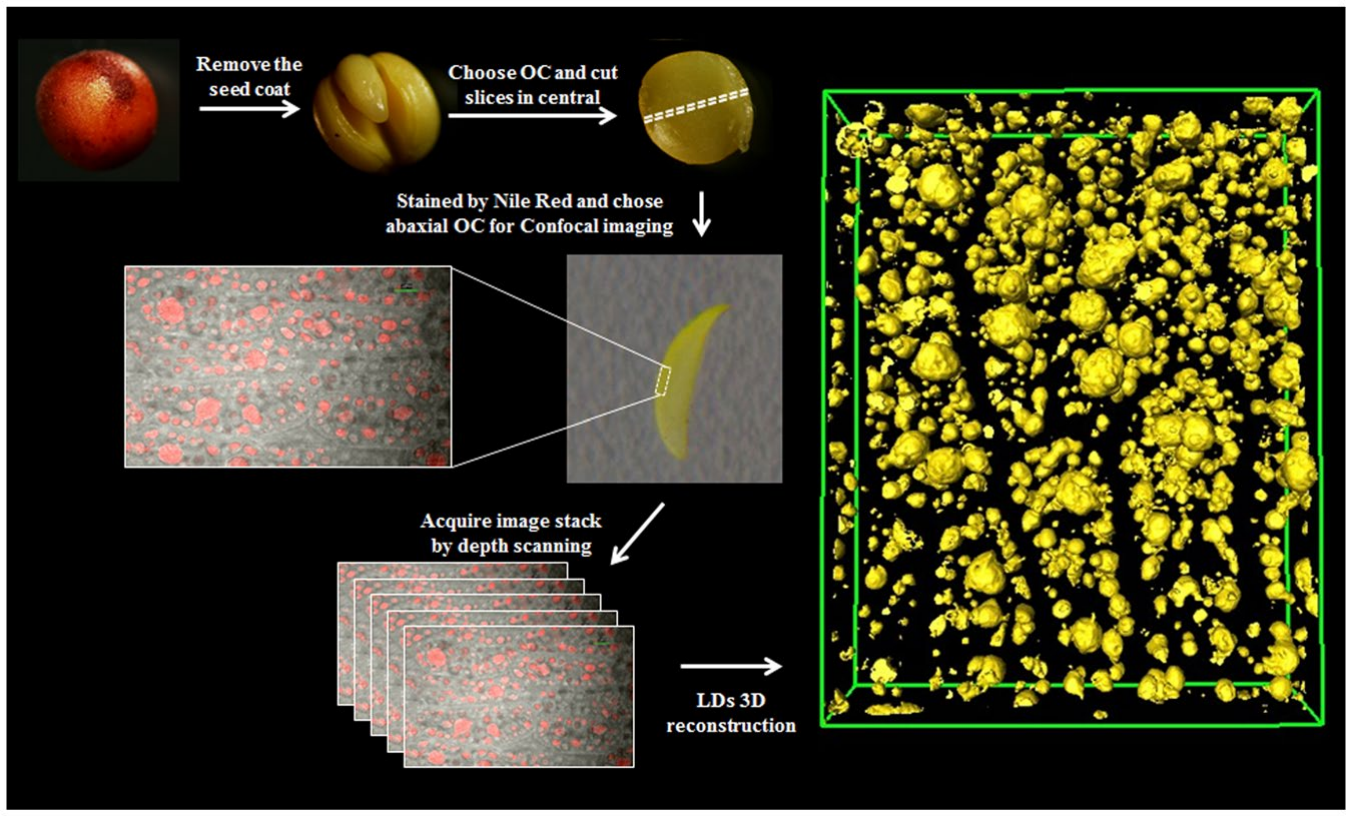
General workflow for LD 3D reconstruction in *B. napus* seed. OC, Outer Cotyledons. Seeds of *B. napus* were prefixed and dissected to obtain outer cotyledons. The central part of the OC was cut into slices and stained with Nile Red to acquire stacks of LD images by confocal microscopy. The image stacks were computed by the AMIRA program via 3D rending and reconstruction. The images shown in the workflow figure were acquired by stereomicroscope and confocal imaging and combined into a single image by Adobe Photoshop software.

**Figure 2:**
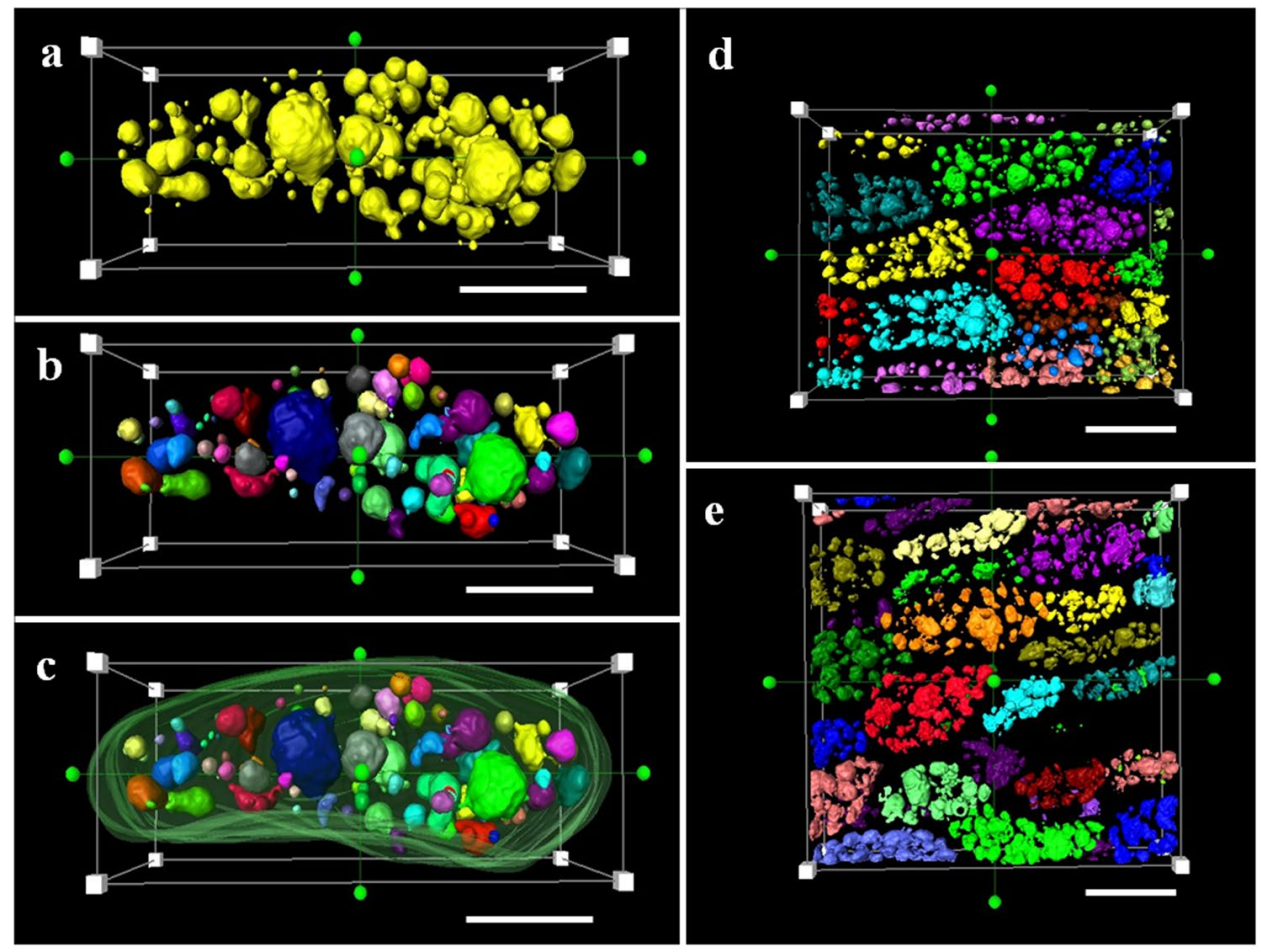
LDs and cell segmentation in 3D level. (**a**) LD segmentation in a single cell; All the LDs in a single cell were segmented by selecting all the LDs voxels as one same label (yellow color) in order to calculate the total volume of LDs in a single cell. (**b**) Single LD segmentation inside the cell; Individual LDs was segmented by selecting individual LDs voxels as different labels (LDs possessed different colors of from each other) in order to calculate individual volume and number of LDs in the cell. (**c**) Cell configuration segmentation; Cell profiles were segmented by selecting cell boundary voxels with the same label (green color) in order to calculate cell volume. Bar represents 20 μm in (**a**–**d**) LD segmentation in the total scanning field of high oil content; different colors represent LDs in different cells. (**e**) LD segmentation in the total scanning field of low oil content cells. Different colors represent LDs in different cells. Bar represents 50 μm in (**d**,**e**).

The oil distribution of individual seeds from high oil (HO) and low oil (LO) lines was detected by NMR (Figure S1). The lipid deposition in the outer cotyledon (OC) and inner cotyledon (IC) were different, and the lipid gradient was also observed within the OC tissue. Seventy slices from the central part of the OC were acquired by CLSM according to the Nile Red fluorescence and white field signals (Figs 1 and S2, Supplement Movie 1). The 3D structure of LDs and cell shape was acquired according to the 2D stack image by AMIRA (Fig. 1). To measure the total volume of LDs in each cell, the volume of single LDs and the cell volume at the 3D level were analyzed, and cell and LD outlines were marked and segmented. All LDs in single cells were segmented and marked in the same color and reconstructed according to the signal from Nile Red at the 3D level to compute the LD volume in each cell (Figs 2a and S4b). Each LD was segmented and marked with different colors to compute the volume of each LD in a single cell (Figs 2b and S5a). For the purpose of calculating the volume of cells, cell profiles were marked and reconstructed (Figs 2c and S4c). All the LDs in the total scanning field were marked to compute the total volume of LDs in LO and HO lines (Figs 2d,e and S4a, Supplement Movie 2).

### Lipid distribution and structure of HO and LO at the 3D level

**Figure 3:**
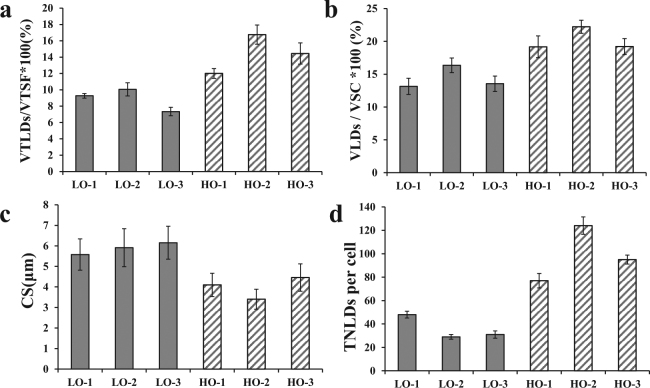
Morphological characteristics of LDs show differences between HO and LO lines. To evaluate the lipid distribution in cotyledons and single cells, the volume of total LDs (VTLDs) and single cells (VSC), as well as the total volume of scanning field (VTSF), was calculated by the AMIRA program; (**a**) VTLD/VTSF value in HO and LO lines; The values of VTLDs/VTSF in HO1, HO2 and HO3 were 12 ± 0.60%, 16.75 ± 1.17%, and 14.44 ± 1.30%, respectively. LO1, LO2 and LO3 were 9.26 ± 0.28%, 10.05 ± 0.80%, and 7.33 ± 0.51%, respectively (n = 10). (**b**) VLD/VSC value in HO and LO lines; Intervals between cells were measured according to the reconstructed cell configuration in 3D level; The values of VLDs/VSC in HO1, HO2, HO3 were 19.17 ± 1.66%, 22.23 ± 1.00%, and 19.22 ± 1.21%, respectively. LO1, LO2, LO3 were 13.15 ± 1.23%, 16.36 ± 1.12%, and 13.55 ± 1.17%, respectively (n = 26). (**c**) CS value in HO and LO; the values of CS in HO1, HO2 and HO3 were 4.10 ± 0.57, 3.40 ± 0.49 and 4.46 ± 0.67 μm, respectively. LO1, LO2 and LO3 were 5.58 ± 0.76, 5.91 ± 0.92 and 6.15 ± 0.80 μm, respectively (n = 10). (**d**) Total numbers of LDs per cell in HO and LO cells; the values of TNLDs per cell in HO1, HO2 and HO3 were 77 ± 6.16, 124 ± 7.44, and 95 ± 3.80, respectively. LO1, LO2 and LO3 were 48 ± 2.88, 29 ± 2.03, and 31 ± 3.1, respectively (n = 10). Values are means ± SD of measurements from HO and LO lines.

**Figure 4:**
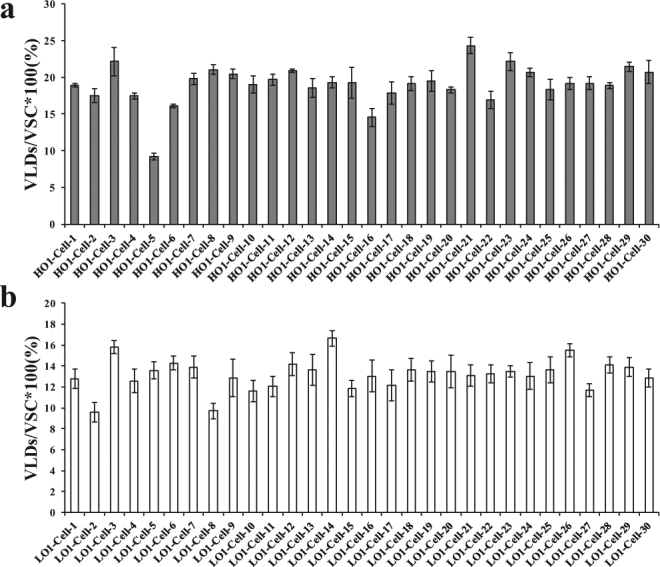
VLD/VSC values in single cells in HO-1 and LO-1 lines. The VLD/VSC values in 30 individual adjacent cells in HO-1 and LO-1 were analyzed and showed that the lipid storage was different among neighboring cells even though they were located in the same tissue. VLDs, volume of LDs in single cells; VSC, volume of single cells; The VLD and VSC values were calculated by the AMIRA program. Values are means ± SD of measurements on VLD/VSC from HO and LO lines (n = 5).

**Figure 5:**
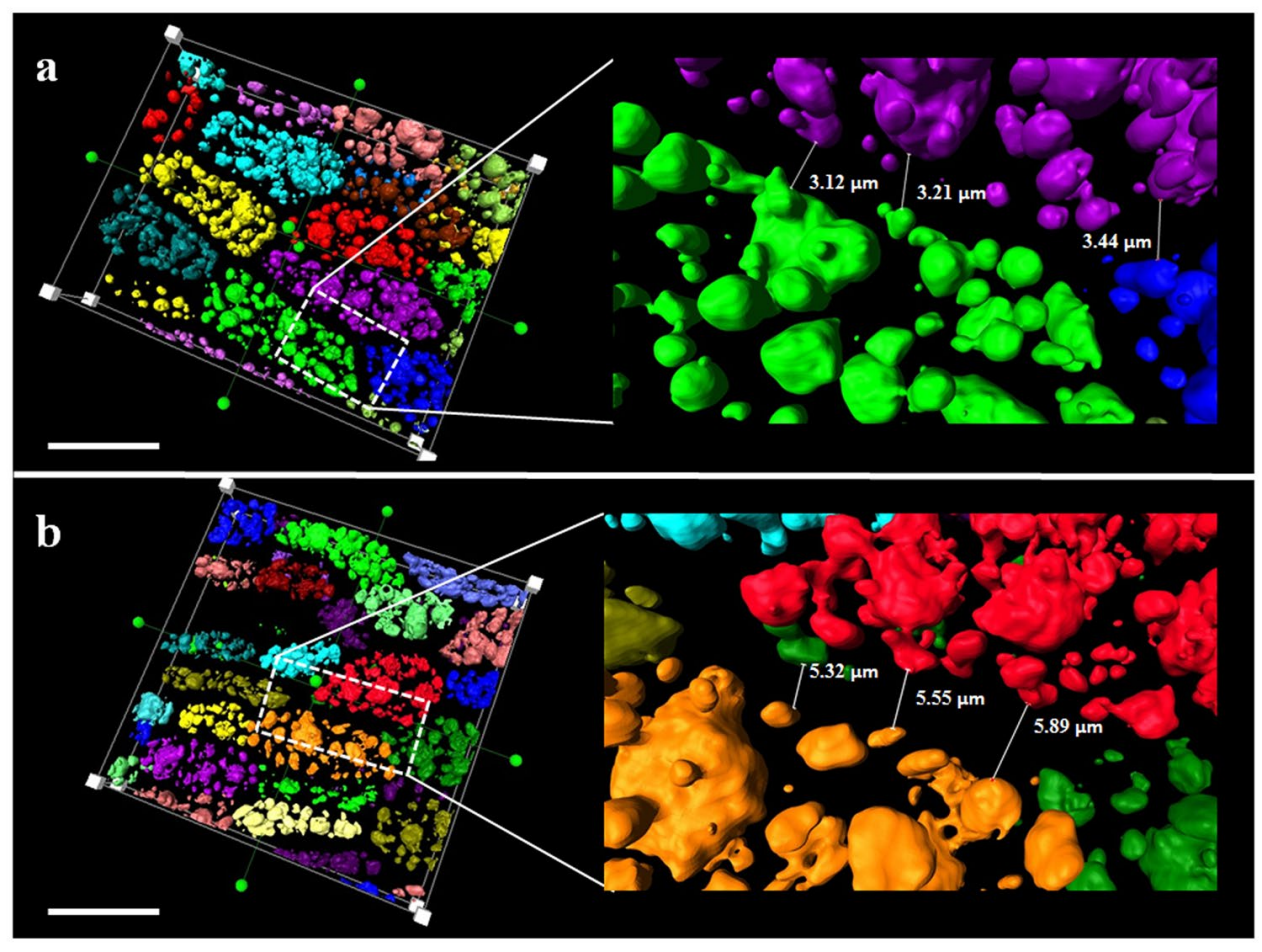
Measurement of cell space in HO and LO line seeds. Intervals between cells were measured according to the reconstructed cell configuration in 3D level; (**a**,**b**) cell space measurement in HO-1 and LO-1 lines; rectangle areas were magnified. LDs in HO and LO line seeds were reconstructed, and the space between LDs in each cell was measured according to the AMIRA 3D measurement tool. Bar represents 50 μm.

To evaluate the lipid distribution in cotyledons and single cells, the volume of total LDs (VTLDs) and single cells (VSC), as well as the total volume of scanning field (VTSF), was calculated using the AMIRA program. The values of VTLDs/VTSF in the three HO materials 09QT181 (HO1), 14356 (HO2) and 09QT328 (HO3) were 12 ± 0.60%, 16.75 ± 1.17%, and 14.44 ± 1.30%, respectively; otherwise, the values for the three LO materials 09QT50 (LO1), 09QT145 (LO2) and 09QT347 (LO3) were 9.26 ± 0.28%, 10.05 ± 0.80%, and 7.33 ± 0.51%, respectively (n = 10, P < 0.01) (Fig. 3a). The volume of LDs in single cells (VLDs) was also calculated by assigning different color markers and integrated separately by AMIRA; the value of VLDs/VSC were 19.17 ± 1.66%, 22.23 ± 1.00%, and 19.22 ± 1.21% in HO lines and 13.15 ± 1.23%, 16.36 ± 1.12%, and 13.55 ± 1.17% in LO lines, respectively (n = 26, P < 0.01) (Fig. 3b). The VLD/VSC values in 30 individual adjacent cells were analyzed. The most interesting phenomenon was that VLD/VSC values varied greatly in different cells even though the observed cells were located in the same tissue or field of vision, and some individual cells showed nearly 2 times higher values than the mean value. In the HO-1 group, some of the individual cells possessed a lower VLD/VSC value than the mean value of LO lines, while the adjacent cells possessed a higher VLD/VSC value (Fig. 4a). The phenomenon mentioned above could also be observed in LO lines, where some individual cells possessed higher VLD/VSC values than ambient cells (Fig. 4b). Those results indicated that the lipid storage of the cells in seeds either in HO or LO lines differed from each other even though they were located in the same tissue. Further analysis revealed that the VLD/VSC value is higher than the VTLD/VTSF value, which indicated that the cell space or LD density showed a highly significant difference in the HO and LO groups. Intervals between cells were measured according to the reconstructed cell configuration at the 3D level. In HO lines, the intervals between cells were 4.10 ± 0.57, 3.40 ± 0.49 and 4.46 ± 0.67 μm, with an average of 3.99 ± 0.48 μm in observed fields of seed (n = 10, P < 0.01). In LO lines, the cell space distances between LDs were obviously higher than that of HO lines, and the space distances were 5.58 ± 0.76, 5.91 ± 0.92 and 6.15 ± 0.80 μm, with an average of 5.88 ± 0.26 μm (n = 10, P < 0.01) (Figs 3c and 5). These results revealed that the cell space distance in the HO group was shorter than that of the LO lines. This result was verified by TEM imaging analysis, the seeds with lower oil content showed thicker cell walls than HO lines (Figure S3). The present results indicate that the intercellular matrix between each cell in LO lines were fuller than in the HO group. From these findings, the oil content level was determined not only by LD density but also by the intercellular matrix between each cell.

**Figure 6:**
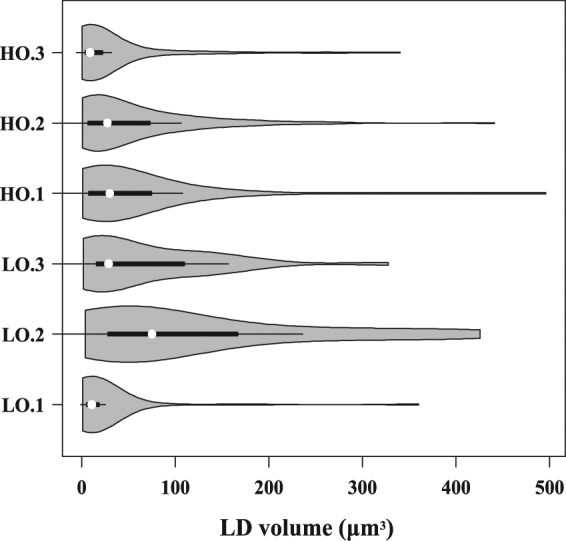
LD size and amounts per cell in LO and HO lines. Total size and amounts of LDs in each line were counted according to the segmentation of single LDs at the 3D level; A box plot and a kernel density plot were generated as violin plots for different groups. The high oil content lines (HO) were 09QT181 (HO-1), 14356 (HO-2) and 09QT328 (HO-3). The low oil content lines (LO) were 09QT50 (LO-1), 09QT145 (LO-2) and 09QT347 (LO-3).

The total numbers of LDs (TNLDs) in each cell were also counted according to the segmentation of single LDs at the 3D level (Fig. 2b). In LO lines, the TNLDs per cell were 48 ± 2.88, 29 ± 2.03, and 31 ± 3.1; otherwise, the TNLDs per cell in HO lines were much higher at 77 ± 6.16, 124 ± 7.44, and 95 ± 3.80 (Fig. 3d). LD size and its number were also calculated and showed that LDs (volume less than 100 μm^3^) occupy a high percentage of total LDs in each cell (Fig. 6). Unusually large LDs (diameter over 5 μm) could be found in both HO and LO lines. LO-2 occupied a higher proportion of unusually large LDs (55%) in total LDs. The other two lines LO-1 and LO-3 in the LO group showed 6% and 35% unusually large LDs, respectively, while the HO group showed 27%, 29% and 11% (data not shown). The average numbers of unusual large LDs in each cell were 3 ± 0, 11 ± 1 and 16 ± 1.3 in LO lines, while HO lines were 20 ± 1.1, 37 ± 0.5 and 11 ± 0.5 (data not shown).

## Discussion

Oil content is one of the most concerned factors by scientists dedicated to elevating oil production in oilseeds in the past decades. Most of the research on seed oil content was conducted at the individual seed level by gas chromatography (GC), near-infrared^36^ and NMR. The relative oil content in OC, IC and hypocotyl differed according to the NMR image in oilseed^23–25,37,38^. A pronounced lipid gradient was established within the OC and within the embryo axis, with two-fold concentration differences within distances of only 300 μm^25^. Recent studies revealed that the spatial distribution of two major lipid species, triacylglycerols and phosphatidylcholines, demonstrated significantly different lipid compositions between the various tissue types within the seed by laser-based mass spectrometry imaging^39^. Those findings acquired by newly applied technology showed that the oil distribution in individual seeds was different even in the adjacent tissues. The reason why oil distribution was different among various tissues proved that light^25^, air exchange^40^ and nutrient transport may affect the oil accumulation inner of seeds, but the mechanism was still unclear. It can be predicted that research focusing on seed oil content will draw more attention at the cell or tissue level in the near future.

Transmission electron microscopy (TEM) has been widely used to observe LDs and lipid distribution in *B. napus* seeds. With the aim of measuring the exact size of LDs, TEM methods might not accurately estimate the volume of cells or LDs because the shape of LDs and cells in seeds were spheriform or irregular spheres. The diameter of the cell or LDs acquired by TEM only represents the diameter of the circle on the surface of the sections and might not reflect the veritable volume size of the cell or LDs, as TEM had been reported to be prone to erroneous in the merging of images^41^. The statistical data in cell or LD size measurements according to TEM imaging may cause inaccuracy if the number of cells or LDs counted in the surface of sections were not sufficient. Additionally, if the shape of the cell or LDs is irregular spheres, it will be complicated to calculate the actual volume. There was no effective technology to acquire the exact cell and LD volume in individual cells, which limited the further study on elevating the oil content in seed.

In the present study, the configuration of cells and LDs in seeds was obtained in *B. napus* by confocal imaging combined with 3D reconstruction technology. The configuration of cells and LDs in the seed can be observed in stereo and can be calculated at an accurate level with the help of the AMIRA program. Although the slices were acquired by slicing technology, the scanning imaging of cells and LDs was still integrated due to laser penetration, avoiding mechanical damage. Tissues in seeds were cut into slices with a thickness of 0.5 mm, while the laser signal could penetrate up to 26 μm, which is exactly the thickness of one layer of cells in seeds (Figure S1). In this way, the shape of the cell and LDs at the 3D level was non-destructive based on CLSM compared with other reconstruction methods depending on the mechanical separator slicer^2,42,43^. Utilizing the high distinguishability of the CLSM 100× oil immersion lens, the cell and LD shape could be clearly distinguished. Eight to ten integrated cells could be observed in the scanning field. Based on the volume of LDs and cell voxels at the 3D level, the actual volume of each LD and cell was calculated. In this way, the relative oil content in each cell could be acquired by the ratio of VLDs to VSC. Based on the 3D structure, cell spaces and LD numbers could also be easily acquired by the measurement tool of the AMIRA program (Figs 2 and 3). CLSM-based 3D reconstruction of LDs and cells in *B. napus* seed enables the detection of single cell oil content and paves a way to improve oil content at the single cell level.

Increasing the oil content in oilseed crops is one of the major obstacles for breeders. LDs were of interest to scientists because of their relationship with levels of oil content^28–30^. Correlations between the quantity and the sum of areas of LDs and oil content were concerned by many scientists^34,44–47^. We succeeded in segmenting the single LDs in each cell and cell membrane computed by AMIRA, and single LDs and cell volume were obtained. Cell volume was acquired according to the segmentation of the cell membrane in the bright field. LD volume was also acquired based on the segmentation of LD shape profiles in the fluorescence field. The VLD/VSC value was significantly higher in HO lines than in LO lines. These results revealed that the VLD/VSC value was positively correlated with high oil content. The VLD/VSC value also reflects the density of LDs in each cell. This was the first observation of the LD density at the 3D level that agreed with previous studies that LDs in the low oil content group were loosely arranged and mostly elliptically shaped in *B. napus*^47^.

There are many indexes such as size, number and density of LDs that might affect the oil content in seed^31,44^. Some scientists predicted that the maximum rapeseed oil content could reach 75% based on the structural analysis of different oil content rapeseed^44^. It will be important to make the seed structure and characteristics of rapeseed clear to achieve a promising goal in breeding. Based on the 3D construction of LD of the seed, the intervals of each cell were measured and compared in HO and LO lines. Our observation revealed that the cell space distance in the HO group was shorter than in the LO lines, which implied that the intercellular matrix in the LO group between each cell was fuller than the higher oil content group. Based on this finding, the oil content level was determined not only by LD density but also by the intercellular matrix between each cell. The present new finding might offer a new index to evaluate the oil content in seeds. Certainly, this finding requires further confirmation in more rapeseeds in the future.

For a long time, few reports have been found referring to the single cell oil content in seeds at the 3D level. Due to technology limitations, research on seed oil content has mainly focused on whole integrated seed instead of tissue level. In this work, we succeeded in evaluating the oil content in single cells in seeds. The values of VTLD/VTSF and VLD/VSC were introduced to evaluate the oil content in seed tissue and single cells that had been applied in monitoring cell metabolism and lipid production in human adipose tissues^48^. The value of VLD/VSC in a single cell was found to be significantly different in each cell even in the same adjacent outer cotyledon. In HO-2 lines, five integrated cells in a 127 μm × 127 μm area were observed with VLD/VSC values ranging from 15% to 27%. Similar observations could be found in five other high or low oil content lines. Those findings were new discoveries and were not mentioned in the previous studies due to the limitation of measurement at the 2D level. In recent years, with the utilization of NMR, scientists have revealed tissue variance in lipid deposition^25,37,38^. The oil content of the outer cotyledon is higher than that of the inner cotyledon and hypocotyl within one seed. The tissues located in different parts of the outer cotyledon, such as palisade tissue and spongy tissue, possessed dramatic differences in oil content from each other^25^. Even the fatty acid component of triacylglycerol and phosphatidylcholine in the inner and outer cotyledon was also different from each other^39^. Those phenomena were confirmed in the six selected lines that the oil distribution in OC or IC was different and showed a pronounced lipid gradient within the OC according to the results of nuclear magnetic resonance (NMR) detection in the seed of our ongoing work (Figure S1), as well as previous descriptions^49^. The present finding shows that cell oil contents in adjacent areas from the same tissue are different, which will offer further understanding of differential cell populations and provide a new way to enhance seed oil content within seed.

LD size is one of the most important characteristics that might affect oil content in seeds^26,27,50,51^. With the help of the segmentation module in the AMIRA program, we succeeded in segmenting LDs and calculating the volume of LDs. There has been a controversy for many years whether ultra-unusual large LDs correlated with higher oil content. TEM imaging in maize seed revealed that the oil bodies in high oil content lines were larger and had a spherical shape, whereas those in low oil content lines were smaller and had irregular shapes^52^. Similar results were found in more than fifteen breeding lines in *B. napus* that seeds in high oil content lines had larger LDs and lined up tightly^47^. Other researchers found that unusually large oil bodies (OBs) are highly correlated with lower oil content in *B. napus*^34^. According to the present observation, unusually large LDs could be found in both HO and LO lines and LD numbers in each cell were significantly higher in HO lines than LO lines (Fig. 6). Our findings are in agreement with a previous study that showed that the higher oil content cultivars possessed more LDs in *B. napus*^45,47^.

In this work, we reconstructed the 3D configuration of neutral lipids and cells in high and low oil content *B. napus* seeds. Features of neutral lipids in high and low oil content *B. napus* seeds were described for the first time at the 3D level. In seeds with high oil content, the cell internal space was smaller, and there were more LD numbers in single cells than in seeds with low oil content. The new finding that the oil content of cells in adjacent areas from the same tissue is different will offer further understanding of differential cell populations and provide a new perspective to enhance the seed oil content at the cell level within seeds.

## Materials and Methods

### Plant Material and Growth Conditions

The *B. napus* lines 09QT181, 09QT328, 09QT50, 09QT145 and 09QT347 were collected from the DH population derived from the cross of Ken C8 and N53-2, and 14356 was collected from 13yhj49-2. The high oil content materials were 09QT181 (HO1), 14356 (HO2) and 09QT328 (HO3), with oil contents of 56.83%, 60.37% and 56.87%, respectively. The low oil content materials were 09QT50 (LO1), 09QT145 (LO2) and 09QT347 (LO3), with oil contents of 33.00%, 34.49% and 36.00%, respectively. The six lines were high erucic acid rapeseed (HEAR) and winter varieties cultured under field conditions in Wuhan, China.

### Fixation and Section of Seed Tissues

For the staining of LDs in matured dry seed, the seeds were fixed for 24 hours in 2.5% glutaraldehyde in a 0.1 M phosphate buffer, pH 6.8, accompanied by vacuum pumping. The fixed seeds were dissected under a SMZ1000 stereomicroscope (Nikon, Japan), and the OC was chosen to cut into slices in the HO and LO groups to ensure that the observation area was equal. Transversely sliced sections in the central part of the OC were selected to make the analysis sites of tissue relatively the same in different samples. The central part, or scilicet “arch crown” of the slice, was chosen for CLSM imaging. The slices were transferred for incubation in Nile Red phosphate buffer solution (1 μg/ml) for 10 minutes to visualize the neutral lipids^53,54^. Only the middle slices in the outer cotyledons were collected to ensure that all of the sections were from the same tissue sites. After staining, the slices were washed in 0.1 M phosphate buffer, pH 6.8, 3 times to remove the residual Nile Red. The samples were then transferred onto microscope slides and covered with a coverslip.

### Confocal Imaging

Images were acquired using an inverted confocal laser microscope FV1000 (Olympus, Japan) using a 100 × 1.4 NA objective. Fluorescence labeling from Nile Red was observed with a 559 nm excitation wavelength and an emission wavelength from 570 to 670 nm. The images in X-Y dimensions were 800 × 800 pixels with sizes of 127 μm × 127 μm, 0.159 μm per pixel in X-referential and 0.159 μm per pixel in Y-referential. In total, 70 images ranging from 25–30 μm in depth were collected, and the distances of each slice were 0.38 μm from each other. The staining and imaging conditions of different samples were controlled to be coordinative.

### 3D Reconstruction and Rendering

For optimal visualization of the tiny architecture of LDs, the raw image data were preprocessed to remove the noise and non-uniform brightness as described previously^12^. The preprocessed data in stacks were then reconstructed in three dimensions and rendered with the use of the program Amira 5.4.0 to show the LDs in whole sections^12^. To distinguish different cells in seeds, each cell membrane profile was appointed in different colors, and the cell volume was calculated using the Amira program. The value of each cell volume was then multiplied by 1.11 × 10^2^ μm^3^ to obtain the actual cell volumes (each pixel in space represents 1.11 × 10^2^ μm^3^). The LDs in the image stacks were segmented according to the boundary between different cells and reconstructed at the 3D level. The total volume of LDs in each cell was calculated as described in the cell volume.

### Transmission Electron Microscope Imaging

Seed using for TEM section were prefixed in 2.5% glutaraldehyde in 0.1 M phosphate buffer, pH 6.8, overnight with vacuum pumping. The prefixed embryos were stained and handed for imaging under an H-7650 transmission electron microscope (Jeol, Japan). The equipment and settings parameters were the same according to a previous description^44^.

### Imaging of *B. napus* seed by NMR

MRI experiments were performed on a Bruker 750 MHz Avance system (Bruker Biospin, Rheinstetten, Germany) in accordance with a previously described method^23^.

### Data availability

No datasets were generated or analyzed during the current study.

## Electronic supplementary material

Supplemental information

supplement movie 1

supplement movie 2
