# Supplementary material for: 3D Reconstruction of Lipid Droplets in the Seed of Brassica napus
Source: Sci Rep. 2018 Apr 26;8:6560. doi: 10.1038/s41598-018-24812-2 (PMC5920073; doi:10.1038/s41598-018-24812-2)
Supplement: Supplementary file 1 — Supplemental information [file 41598_2018_24812_MOESM1_ESM.pdf]

3D Reconstruction of Lipid Droplets in the Seed of *Brassica napus*

Yongtai Yin[1,3], Liangxing Guo[1], Kang Chen[1], Zhenyi Guo[1], Hongbo Chao[1], Baoshan Wang[2], Maoteng Li[1,3,*]

[1] Department of Biotechnology, College of Life Science and Technology, Huazhong University of Science and Technology, Wuhan, 430074, China

[2] College of Life Science, Shandong Normal University, Jinan, 250000, China

[3] Hubei Key Laboratory of Economic Forest Germplasm Improvement and Resource Comprehensive Utilization, Hubei Collaborative Innovation Center for the Characteristic Resources Exploitation of Dabie Mountains, Huanggang Normal University, Huanggang, 438000, China

Supplemental figures

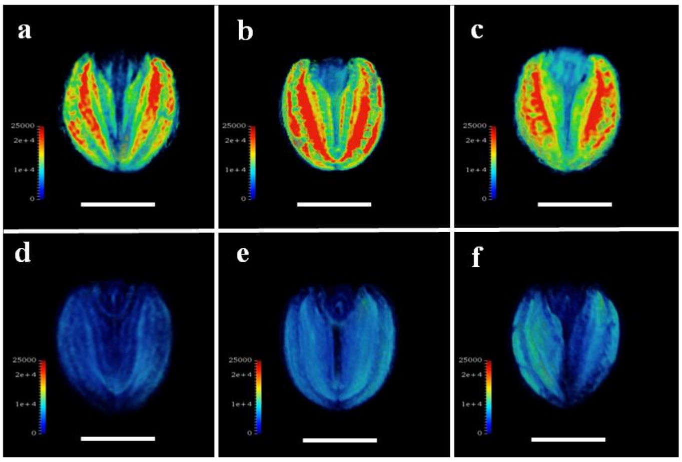

Figure S1 Imaging of *B. napus* seed by Nuclear magnetic resonance (NMR)

A pronounced lipid gradient was established within the outer cotyledon, inner cotyledon and also within the embryo axis. a, b and c were 09QT181, 14356 and 09QT328; d, e and f were 09QT50, 09QT145 and 09QT347. Red color represents the signal intensity of neutral lipid. Bar represents 1 mm. The color scale indicates lipid concentration (red represents high oil deposition and blue represents low oil deposition).

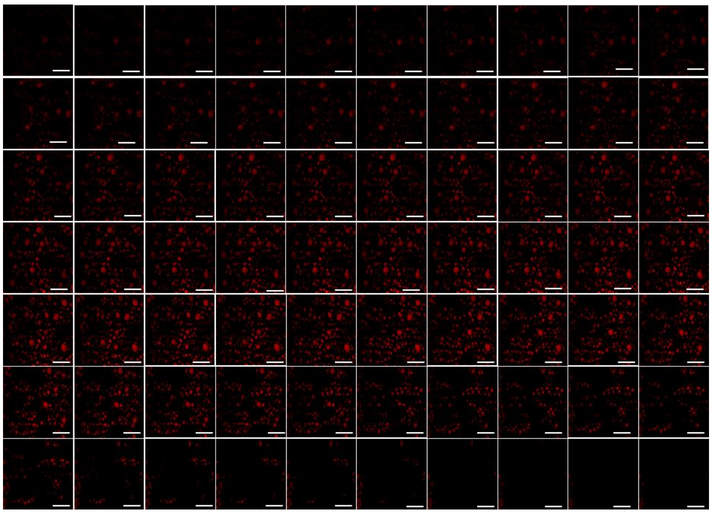

Figure S2 Confocal Depth scanning of Nile red stain signal in stacks

Images were acquired by confocal depth scanning by scanning step in Z-direction 0.38 μm.

Bar represents 30 μm.

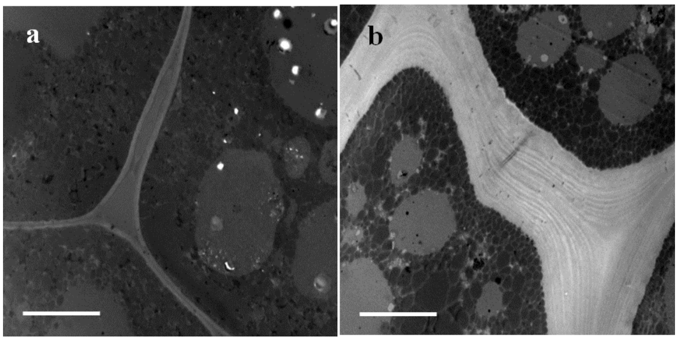

Figure S3 Cell space in HO and LO line seeds detected by TEM

TEM technology was applied to acquire the image of LDs and cell internal space in 2D level. a, represents the HO line seed; b, represents the LO line seed. Bar represents 5 μm.

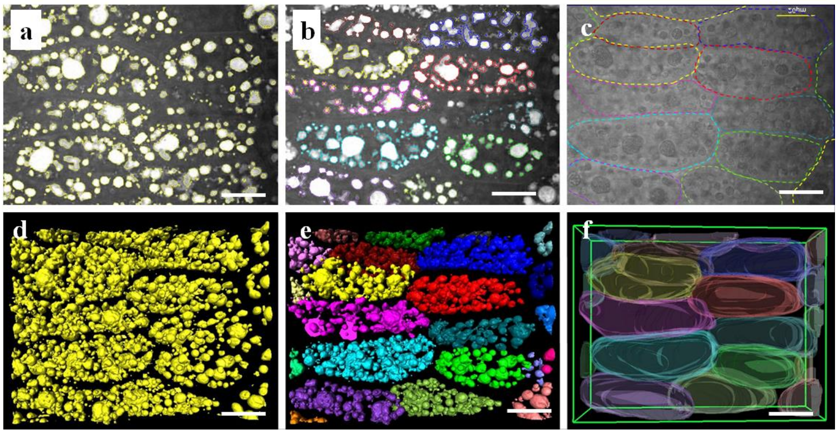

Figure S4 LDs and Cell Segmentation and 3D Reconstruction

a, Segmentation of total LDs in scanning field; b, Segmentation of LDs in each cell; c, Segmentation of cells in scanning field; d, e and f, 3D reconstruction of total LDs in scanning field, LDs in single cell and the cells in scanning field. Bar represents 20 μm.

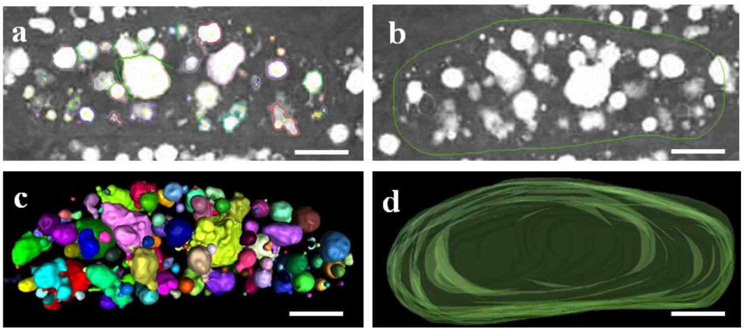

Figure S5 Single LDs in One Cell Segmentation and 3D Reconstruction

a, Segmentation of single LDs in each cell; b, Segmentation of cell outline; c and d, 3D reconstruction of single LDs in one cell. Different color represents different voxel assigned to each single LDs. Bar represents 10 μm.

Supplemental videos

Supplement Movie 1. Image stacks of LDs and cell shape acquired by CLSM.

Supplement Movie 2. Segmentation of LDs and cells in 3D level.
